# Treatment Outcomes in Patients with Polypoidal Choroidal Vasculopathy

**DOI:** 10.4274/tjo.19981

**Published:** 2016-01-05

**Authors:** Işıl Sayman Muslubaş, Mümin Hocaoğlu, Serra Arf, Hakan Özdemir, Murat Karaçorlu

**Affiliations:** 1 İstanbul Retina Institute, İstanbul, Turkey

**Keywords:** Polypoidal choroidal vasculopathy, photodynamic therapy, intravitreal bevacizumab

## Abstract

**Objectives:**

To report outcomes of photodynamic therapy (PDT) and combined therapy with PDT and intravitreal bevacizumab (IVB) in patients with polypoidal choroidal vasculopathy (PCV).

**Materials and Methods:**

Thirty-four eyes of 31 patients with subfoveal PCV were evaluated. Nine eyes were treated with PDT and 25 eyes treated with combined therapy of PDT and IVB. All eyes had a follow-up period of at least 12 months. In this retrospective study the demographic features, best corrected visual acuity, fundus color photography, optical coherence tomography, fluorescein angiography and indocyanine green angiography of the 34 eyes were evaluated.

**Results:**

Visual acuity improved but did not change significantly in the patients treated with PDT and combined PDT+IVB therapy (p=0.149; p=0.087). Although the mean central foveal thickness decreased in both groups, there was no statistically significant difference between groups (p=0.98). The polypoidal lesions regressed in 6 (66.7%) of 9 eyes in the PDT monotherapy group and 16 (64%) of 25 eyes in the PDT+IVB combined therapy group.

**Conclusion:**

Both PDT and a combined therapy of PDT and IVB yielded successful outcomes in patients with PCV.

## INTRODUCTION

Polypoidal choroidal vasculopathy (PCV) is a retinal disorder characterized by aneurysmal polypoidal lesions in the choroidal vasculature. It has been considered a subgroup of age-related macular degeneration (AMD), but the presence of polypoidal lesions beyond the macula and differing responses to treatment suggest it is a separate pathology.^1^ Polypoidal lesions may be visible as red-orange lesions on fundus examination, though the definitive diagnosis is made by indocyanine green angiography (ICGA).^2^ Many studies have reported successful results with subfoveal PCV treatment by photodynamic therapy (PDT), anti-vascular endothelial growth factor (anti-VEGF) therapy, and combination therapy with both PDT and anti-VEGF.^3^

The purpose of this study was to present the treatment outcomes of subfoveal PCV patients who underwent PDT or combined PDT and anti-VEGF therapy.

## MATERIALS AND METHODS

Nine eyes of 9 patients treated with FDT and 25 eyes of 22 patients treated with FDT and intravitreal bevacizumab (IVB) due to subfoveal PCV between 2004 and 2014 were evaluated retrospectively. Patients with previous ocular surgery, cataract development that would affect visual acuity, glaucoma, or other retinal pathologies were excluded from the study.

Initial and follow-up examinations included visual acuity (VA) assessment by ETDRS chart, intraocular pressure (IOP) measurement and examinations of the anterior segment and fundus, followed by fluorescein angiography (FA), ICGA and optical coherence tomography (OCT). Standard PDT was performed as described in our previous study,^4^ after which all patients were followed-up by examinations at 1 month, 3 months, and at 3 month intervals thereafter for at least 12 months. In the combined therapy group, patients underwent standard PDT followed by IVB injections each month for the first 3 months and as needed thereafter as described in our previous study,^5^ and were followed for at least 12 months. The earliest leakage from neovascular structures was observed after 6 months; these patients underwent repeated PDT.

In accordance with the principles of the Declaration of Helsinki, patients were informed about their current status, natural course, treatment success rates and risks, and consent was obtained for all PDT and anti-VEGF treatments.

Patients’ age, gender, number of PDT sessions and anti-VEGF injections and duration of treatment, pre- and post-treatment VA and foveal thickness were evaluated. Recurrences, regression of polypoidal lesions and complications were also reported.

Wilcoxon significance test, Mann-Whitney U test and Pearson’s chi-square test were used in the statistical analyses. The level of significance was accepted as α=0.05.

## RESULTS

Nine patients with subfoveal PCV underwent FDT alone and were followed for a mean of 23.6±13.9 months (range 12-48 months); 2 were women and the average age of the group was 64±7.6 years. Although an increase in mean VA was observed between pre-treatment (0.40±0.27 logMAR) and both 1 year post-treatment (0.25±0.36 logMAR) and at the end of follow-up (0.25±0.36 logMAR), a significant difference in VA could not be detected between the time points, possibly due to the small patient number (p=0.149, p=0.149, respectively). At the end of the mean 24 month follow-up period, VA had increased in 6 (66.7%) patients and remained stable in 2 (22.2%). The VA of one patient declined by two rows according to the ETDRS chart. A decrease in mean foveal thickness was observed by OCT at 1 year post-treatment (256.22±86.73 µm) and after a mean of 24 months follow-up (276.78±142.98 µm) compared to pre-treatment (312.56±61.74 µm); however, the differences were not statistically significant (p=0.236 and p=0.441, respectively). Recurrence was observed in 3 (33.3%) of the patients during the first 12 months of follow-up, in 1 (25%) of the 4 patients followed for 13-24 months, and in 1 (50%) of the 2 patients followed for 25-36 months; no recurrence was observed during months 37-48 of follow-up. Recurrence was observed in 3 eyes (33.3%) and regression of polypoidal lesions was detected by ICGA in 6 eyes (66.7%) during the follow-up of mean 24 months. Cases with recurrence underwent a mean of 3.3±1.4 sessions of PDT in total.

Twenty-two patients (9 women, 13 men) underwent combined treatment with PDT and IVB and were followed for a mean of 24.6±15.4 months (range, 12-48 months); the mean age of the group was 64.8±8.2 years. Patients received an average of 8.3±5.8 IVB injections. A significant difference in VA was found between pre-treatment (0.33±0.32 logMAR) and 1 year post-treatment (0.22±0.29 logMAR) (p=0.025). Although there was also VA improvement between pre-treatment and during follow-up with a mean duration of 24 months (0.23±0.31 logMAR), the difference was not statistically significant (p=0.087). At the end of follow-up, VA had improved in 16 (64%) eyes, remained the same in 6 (24%) eyes and declined by 2 rows on the ETDRS chart in 3 (12%) eyes. There was a significant difference in mean foveal thickness determined by OCT between pre-treatment (349.76±122.09 µm) and both 1 year post-treatment (248.96±87.63 µm) and during the follow-up of mean 24 months (249.8±49 µm) (p=0.001 for both). Recurrence was observed in 4 (16%) of the patients during the first 12 months of follow-up, in 5 (45.4%) of the 11 patients followed for 13-24 months, in 4 (50%) of the 8 patients followed for 25-36 months, and in 2 (50%) of the 4 patients followed for 37-48 months. During the follow-up period lasting a mean 24 months, recurrence was found in 11 eyes (44%) and regression of polypoidal lesions detected by ICGA was found in 16 eyes (64%). Cases with recurrence underwent an average of 2.7±1.1 sessions of PDT.

There were no differences between the two groups in age, gender, and VA or foveal thickness before treatment or after an average follow-up of 24 months (p=0.80, p=0.67, p=0.41, p=0.95, p=0.57, and p=0.98, respectively). None of the patients developed complications related to treatment.

**Figure 1:**
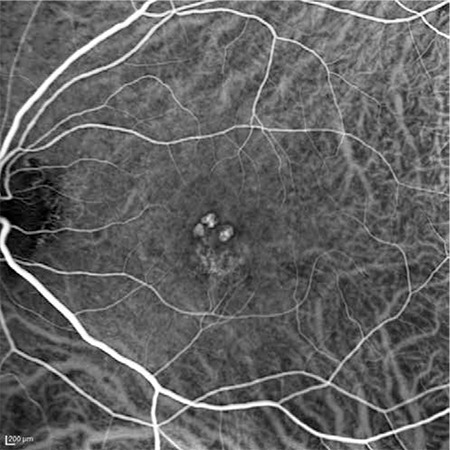
Indocyanine green angiography of a patient with polypoidal choroidal vasculopathy in the left eye showing three subfoveal polyps and an area of hyperfluorescence from a branching vascular network

**Figure 2:**
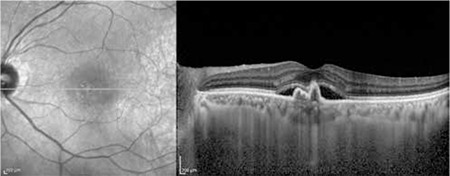
Optical coherence tomography from the same patient showing the polyp from the last section and shallower pigment epithelial detachment due to the brancing choroidal network

**Figure 3:**
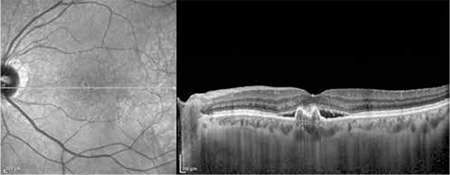
Optical coherence tomography of the same patient at 1 month follow-up, after photodynamic therapy and the first intravitreal bevacizumab injection. Regression of the subretinal fluid in the left eye can be seen, but it is still present in sporadic areas

**Figure 4:**
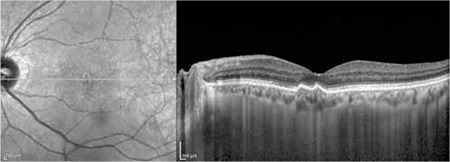
Optical coherence tomography of the same patient at 2 month follow-up, after injection of the second dose of intravitreal bevacizumab. Regression of the subretinal fluid in the left eye can be seen

**Figure 5:**
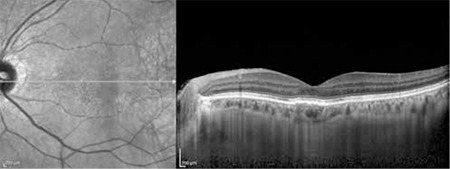
Optical coherence tomography of the same patient at 3 month follow-up, after injection of the third dose of intravitreal bevacizumab. Further regression of the subretinal fluid in the left eye can be seen

**Figure 6:**
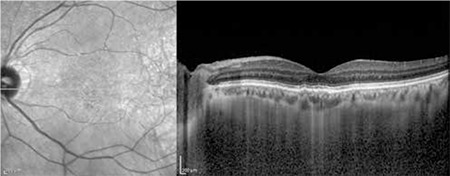
Optical coherence tomography of the same patient at 6 month follow-up; no intraretinal or subretinal fluid is visible in the left eye

**Figure 7:**
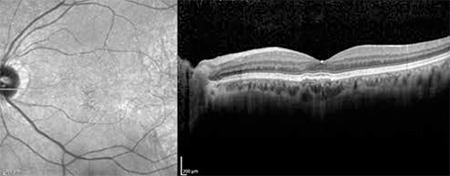
Optical coherence tomography of the same patient at 1 year follow-up. No intraretinal or subretinal fluid is visible in the left eye

The patients’ demographic and treatment characteristics are shown in Table 1. Pre- and post-treatment visual and anatomical outcomes of the PDT and combined PDT+IVB treatment groups are shown in Table 2. Figures 1, 2, 3, 4, 5, 6, 7 show the pre-treatment appearance and post-treatment results at 1, 2, 3, 6, and 12 months of an eye with subfoveal PCV that underwent PDT and received 3 consecutive doses of IVB.

## DISCUSSION

**Figure 8:**
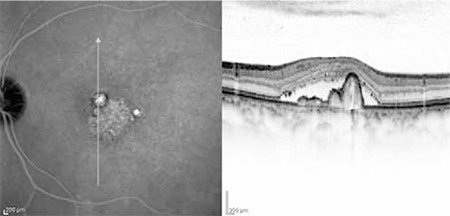
Left: Indocyanine green angiography of polypoidal choroidal vasculopathy patient showing polypoidal formations appearing as vascular aneurysmal dilations of the inner choroidal vessels as well as hyperfluorescence typical of the branching vascular network. Right: Polypoidal choroidal vasculopathy diagnosis is facilitated by optical coherence tomography showing subretinal fluid accumulation, branching vascular network in the form of shallow fibrovascular pigment epithelial detachment, and polypoidal structures within the raised pigment epithelial detachment

PCV presents with polypoidal vascular dilations in the choroidal vasculature and a branching choroidal vascular network. Definitive diagnosis can be made using ICGA, in which polypoidal structures appear as vascular aneurysmal dilations originating from the choroidal vessels and hyperfluorescence from the branching vascular network is visible.^2,6,7^ Retinal pigment epithelial detachment (PED) typical of PCV can be detected with OCT. The visualization of subretinal fluid accumulation, a branching vascular network in the form of shallow fibrovascular PED, and the presence of polypoidal structures within the raised PED on OCT facilitates the diagnosis of PCV, and OCT is also beneficial in monitoring treatment response (Figure 8).^6,7^

Thermal laser coagulation is used to treat extrafoveal polypoidal lesions, but it is not currently recommended for the treatment of subfoveal and juxtafoveal PCV.^6,8^

Many studies have demonstrated that PDT alone has short- and medium-term success rates of 80% and 95% in the treatment of subfoveal PCV.^3^ Gomi et al.^9^ reported that of 36 patients who underwent PDT, VA was increased in 67% and stable in 17%, and polyp regression was achieved in 86% at 1 year follow-up. Şentürk et al.^4^ found VA had increased in 60% and stabilized in 40% of their patients at 1 year after PDT. Otani et al.^10^ also found that after PDT, 82.2% of their 45 patients exhibited polypoidal lesion regression at 1 year follow-up. Similarly, in the current study we found that VA increased in 67% of our patients and stabilized in 22% over a mean follow-up duration of 24 months, and polypoidal lesion regression was detected in 67% of the cases.

Intravitreal anti-VEGF agents have been shown in many studies to be effective in the treatment of choroidal neovascularization associated with AMD, but their efficacy in the treatment of PCV is limited.^5,11,12^ Several studies have shown that anti-VEGF therapy resulted in decreased retinal thickening on OCT and visual and anatomic success; however, polypoidal lesion regression has been achieved through its combined use with PDT or thermal laser coagulation.^11,13^ Although there is evidence of the short-term efficacy of aflibercept (another anti-VEGF agent derived from VEGF receptor) in the treatment of PCV, long-term randomized clinical studies are needed.^14^

There are reports of improved VA and decreased foveal thickness 1 year after combined PDT and IVB injection therapy for PCV.^15^ Similarly, Ruambiboonsuk et al.16 found improved VA and polypoidal lesion regression in all of their cases after combined therapy. During the mean 24 month follow-up period in our study, VA increased in 64% and remained unchanged in 24% of patients who underwent PDT and IVB injection. There was also a statistically significant decrease in central macular thickness at the end of the mean 24 month follow-up period, and polypoidal lesion regression was achieved in 64% of the cases.

Studies have demonstrated that the combination of PDT and IVB injection is as effective as PDT alone in the treatment of subfoveal PCV.^3^ Gomi et al.^17^ compared the results of PDT alone and combined PDT and IVB injection and reported that combined therapy yielded significantly better visual outcomes during the 1 year follow-up period. However, in the randomized controlled study EVEREST, no difference was found between PDT and combined PDT and anti-VEGF therapy in terms of VA improvement or polyp regression (71.4% and 77.8%, respectively), and the rate of polyp regression with anti-VEGF therapy alone was low (28.6%).^13^ Similarly, when we compared the results from our patients treated with PDT or combined PDT and IVB injection during the mean 24 months of follow-up, we found post-treatment anatomic and functional recovery in both groups, and no statistically significant difference emerged in the results.

It has been reported that the visual and anatomic success achieved in the short and medium terms with PDT alone and combined therapy cannot be maintained in the long term.^3^ Because of the high post-treatment recurrence rate, long-term follow-up of these patients is recommended. In our study, additional PDT was administered due to recurrence in 33.3% of cases treated with PDT alone and 44% of those treated with combined PDT+IVB injection. While studies with short follow-up times report approximately 80% visual and anatomical success, our rate was approximately 65%, suggesting that our lower success rate was due to our relatively longer follow-up period.

## CONCLUSION

Both PDT monotherapy and PDT combined with IVB injection yielded successful outcomes in this study. Limitations of this study are that it is retrospective, the study groups had very different patient numbers, and the follow-up period was not long enough. Prospective studies with larger patient numbers and longer follow-up will be useful to demonstrate the long-term treatment results.

## Ethics

Ethics Committee Approval: It was taken, Informed Consent: In accordance with the principles of the declaration of Helsinki, patients were informed about their current status, natural course, treatment success rates and risks, and consent was obtained for all PDT and anti-VEGF treatments.

Peer-review: Externally peer-reviewed.

## Figures and Tables

**Table 1:**
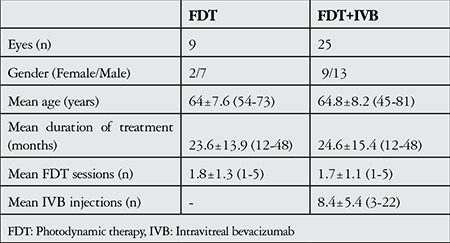
Demographic and treatment characteristics of the patient groups

**Table 2:**
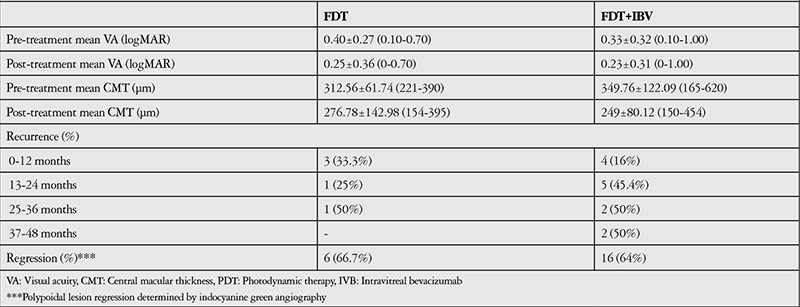
Pre- and post-treatment visual and anatomic outcomes
